# Fostering social inclusion through adapted physical activity: an action research on the psychological experiences of students, teachers, and corporate volunteers

**DOI:** 10.3389/fpsyg.2026.1817674

**Published:** 2026-05-13

**Authors:** Gunhwan Bae

**Affiliations:** Department of Adapted Physical Education, Korea National Sport University, Seoul, Republic of Korea

**Keywords:** action research, adapted physical activity, corporate social responsibility, developmental disabilities, intergroup contact, physical self-efficacy, social inclusion

## Abstract

Inclusive education for students with developmental disabilities remains underserved within conventional corporate social responsibility (CSR) frameworks, representing a critical blind spot in cross-sector social investment. This study explores an innovative collaborative model that integrates corporate resources with expertise in adapted physical activity (APA) to address this gap. Using a two-cycle action research methodology over 11 months, the study analyzed a handball program for students with developmental disabilities involving 35 participants (21 corporate volunteers, 4 project support staff, and 10 special education teachers). Data collected through in-depth interviews and field notes were analyzed using inductive thematic analysis, resulting in 3 core themes: “Cultivating the Ground” (strategic planning), “Blooming the Flowers” (educational growth in students), and “Harvesting the Fruit” (organizational pride and ableism reduction). This study aimed to explore the psychological impacts and behavioral changes of various stakeholders involved. Specifically, it investigates how structured physical interactions foster physical self-efficacy in students with developmental disabilities, and how intergroup contact reduces prejudice and enhances organizational pride among corporate volunteers. Within the specific context examined, the findings suggest that APA-based CSR shows potential as a value-creation model that may promote creating shared value (CSV) by addressing educational blind spots and enhancing social inclusion; however, broader applicability requires further empirical investigation. This research provides a theoretical and practical foundation for expanding sustainable pathways to inclusive education through cross-sector partnerships.

## 1 Introduction

The traditional view of corporate social responsibility (CSR), which primarily prioritized profit maximization (Friedman, 1970), has fundamentally evolved (Aguinis and Glavas, 2012). Today, corporations operate as integral members of a social community, maintaining organic relationships and interacting with a wide range of stakeholders, such as shareholders, employees, customers, and local communities (Freeman, 1984; Carroll and Shabana, 2010). Integrating the resolution of social issues into core management strategies is now essential for cultivating a positive image, securing social legitimacy, and fostering sustainable growth.

CSR is a comprehensive concept encompassing economic, legal, ethical, and philanthropic responsibilities (Carroll, 1991). In this study, CSR is further conceptualized through the lens of social inclusion as defined by the Salamanca Statement (UNESCO, 1994), where corporate engagement acts as a systemic intervention to dismantle educational barriers for marginalized groups, thereby fostering long-term social sustainability (UNESCO, 2005; Shields and Synnot, 2016). For modern enterprises, CSR is no longer an optional endeavor but an essential value that serves as a foundation for long-term survival and growth (Carroll and Shabana, 2010; Crane et al., 2014). Social contribution activities constitute a crucial pillar of corporate CSR (Ahn et al., 2025). Recently, these activities have evolved beyond simple donations or one-time volunteerism toward a strategic direction that leverages a corporation’s unique resources and core competencies to actively address social problems (Porter and Kramer, 2011; Lee, 2022). Although modern enterprises now strive to expand their social contributions by integrating diverse content including educational support, environmental protection, and cultural patronage, these efforts often remain confined to traditional, well-trodden fields (Federation of Korean Industries, 2023).

This concentration of support in certain areas inevitably leaves more specialized fields as “blind spots” in social contribution activities. The field of adapted physical activity (APA) is a representative example of such a blind spot (Yoon and Park, 2022). APA requires a high degree of expertise, which involves a profound understanding of the characteristics of students with disabilities as well as systematic instructional methodologies (Sherrill, 2004). Due to these requirements, APA is often perceived as a challenging area for CSR content, as corporations face significant constraints in planning and executing such programs using only their internal resources (Lee, 2014).

For students with disabilities, participation in APA is not merely about enhancing physical abilities; rather, it represents a vital educational domain that significantly influences social development and the formation of a positive self-concept (Sherrill, 2004). Despite these recognized benefits, systemic environmental and social constraints continue to severely limit the opportunities for students with disabilities to engage in regular, structured physical activities (Shields and Synnot, 2016).

Inclusive education, as articulated in the Salamanca Statement (UNESCO, 1994) and further elaborated in subsequent international guidelines (UNESCO, 2005), refers to a systemic reform ensuring that all learners, including those with disabilities, can participate meaningfully in educational environments. The present program operationalizes this principle by expanding structured physical education (PE) opportunities for students with developmental disabilities within special school settings. Particularly in South Korea, although inclusive education is legally and institutionally promoted (Kwon, 2005), a cultural context persists where segregated special schools are preferred for the tailored education and safety of students with developmental disabilities (Goo and Lee, 2016). While these special schools provide a specialized educational environment, they structurally face chronic shortages of special education teachers and infrastructure limitations (Oh, 2008), making it difficult to operate diverse PE programs independently. In this context, Company S’s handball education support project for students with developmental disabilities in special schools, which is the focus of this study, is noteworthy. It represents a novel collaborative CSR model that integrates the human and material resources of a major corporation, the APA expertise of K University, and the educational environment of special schools. Accordingly, this study aims to conduct an in-depth analysis of this case by employing an action research methodology (Carr and Kemmis, 1986). Action research is a systematic, cyclical inquiry method that integrates research, action, and reflection, enabling researchers to simultaneously contribute to practical problem-solving and generate new theoretical understanding (Carr and Kemmis, 1986; McNiff, 2013). It is particularly well-suited to complex educational settings where the researcher is also a participant in the phenomenon under study (Kemmis et al., 2014). This approach allows the researcher to participate directly in the field, contributing to problem-solving and program improvement, while exploring the underlying significance of the initiative.

The purpose of this study is to present an innovative and sustainable model for inclusive education that merges APA with CSR, establishing a practical and theoretical foundation for its future development. In doing so, this study adopts a psychological lens to illuminate the internal mechanisms of change, drawing upon self-efficacy (Bandura, 1977) and intergroup contact theory (Allport, 1954) to explain how cooperative physical activities dismantle psychological barriers and foster social inclusion. To this end, an action research approach was adopted to analyze the planning and execution process of the program and to explore the experiential meanings of the participants within the context of social sustainability and inclusive education. This study makes three distinct contributions. Theoretically, it provides an empirical application of self-efficacy theory (Bandura, 1977) and intergroup contact theory (Allport, 1954) within an APA-CSR context, extending both frameworks to a novel cross-sector setting. Methodologically, it demonstrates the utility of two-cycle action research as a framework for evaluating community-based CSR programs in educational settings. Practically, it proposes a replicable triadic collaboration model (corporation–university–special school) that can serve as a template for future inclusive education initiatives. The research questions addressed are as follows:

First, what are the characteristics of the planning and execution process of corporate social contribution activities based on APA?

Second, what psychological and developmental significance does the APA-based CSR program hold in the special school field, particularly for students and teachers?

Third, how does participation in the APA-based CSR program influence the psychological perceptions, intergroup attitudes, and organizational pride of corporate members?

## 2 Materials and methods

### 2.1 Action research

This study applied action research, a form of reflective inquiry that integrates theory and practice by having the researcher participate in the problem-solving process as a practitioner in the field (Carr and Kemmis, 1986). The most prominent characteristics of action research are its field-oriented nature and its practical purpose; it is an interpretive process aimed at describing and understanding how participants assign meaning to reality within a specific context (Kim, 2013). This qualitative approach is particularly suitable for specifying research subjects and conducting in-depth investigations within vivid educational settings (Eisner, 1985).

**FIGURE 1:**
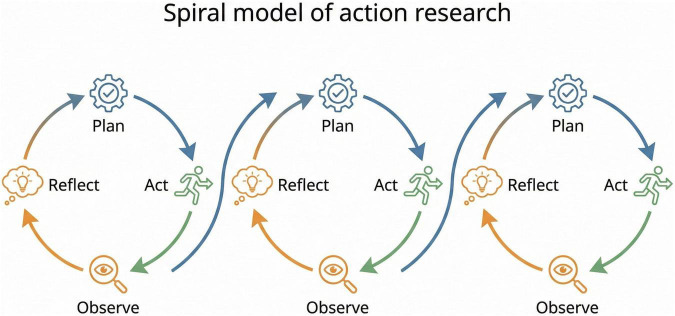
Spiral model of action research.

Since it was first proposed by Lewin (1946), action research has been further developed by various scholars (Carr and Kemmis, 1986; McNiff, 2013). In this study, the research procedure was conducted according to the cyclical spiral model proposed by Kemmis et al. (2014). This model provides a procedural framework ideal for improving various issues encountered in educational fields, characterized by a spiral process of progressive refinement where a cycle of Planning, Acting, Observing, and Reflecting stages leads to the planning of the subsequent cycle (Kemmis et al., 2014; Figure 1). Based on this cyclical spiral model, the present study was conducted over a total of two cycles to ensure both the stable implementation of the program and a comprehensive, in-depth analysis (Table 1).

**TABLE 1:** Spiral model for APA-based CSR program.

Research phase	Cycle 1: program introduction and initial implementation	Cycle 2: program deepening and improvement
Plan	● Establish pre-education plan ●Develop initial program draft Set initial research questions	●Modify and supplement program content ●Review detailed procedures
Action	●Conduct pilot program ●Hold mid-term meetings	●Implement refined program ●Hold mid-term meetings
Observation	●Participate as instructor ●Conduct regular monitoring	●Participate as instructor ●Conduct regular monitoring
Reflection	●Conduct in-depth interviews ●Analyze performance ●Discuss improvement plans	●Hold final stakeholder meetings ●Perform comprehensive analysis ●Draw conclusions

### 2.2 Research participants

In this study, participants were selected using purposive sampling, which is a method of intentionally selecting individuals who can best fulfill the research objectives. The participants consisted of individuals involved in a handball education program conducted at two special schools located in City A. Accordingly, the final selection of research participants included 21 corporate volunteers from Company S, 4 project support personnel including CSR managers, and 10 special education teachers (Table 2).

**TABLE 2:** Characteristics of research participants.

Category	Affiliation	Participants (*N*)	Number of program sessions
Corporate volunteers	Corporation	1–13 (*n* = 13)	1–4
	Corporate handball team	14–21 (*n* = 8)	1–2
Support staff	CSR team	22, 23 (*n* = 2)	4–7
	Project support NGO	24, 25 (*n* = 2)	6–7
Special school teachers	Special school A	26–31 (*n* = 6)	Regular participation
	Special school B	32–35 (*n* = 4)	Regular participation

### 2.3 Data collection

This study was conducted through a two-cycle action research process over approximately 11 months, from September 2024 to July 2025. The first cycle consisted of 9 sessions at School A, while the second cycle involved 14 sessions at School A and 15 sessions at School B. Throughout the research process, qualitative data were collected from multiple sources to ensure data richness, specifically: (1) semi-structured in-depth interviews, (2) the researcher’s field journal maintained throughout all sessions, and (3) program-related documents including volunteer activity logs, session planning records, and CSR team meeting notes. To explore the meanings and experiences of the program in depth, semi-structured in-depth interviews lasting approximately one hour were conducted with corporate volunteers, special education teachers, and CSR managers. These interviews were recorded and transcribed with the participants’ informed consent. The researcher served as the program instructor and participated in the entire process, maintaining a detailed field journal. To ensure neutrality and mitigate potential bias stemming from this dual role, the researcher engaged in constant reflexivity (Finlay, 2002), cross-referencing field observations with support staff feedback and conducting peer-debriefing sessions to validate that the interpretations remained grounded in the participants’ lived experiences. Furthermore, program-related documents were systematically collected and reviewed, including session plans, volunteer activity logs, CSR team meeting minutes, and official program reports provided by Company S. These documentary sources served to triangulate the interview data and field observations. The operational details of the program implemented in this study are summarized in Table 3.

**TABLE 3:** Details of the APA-based CSR program session.

Time	Program phase and content	Instructor role	Volunteer role
30 mins	[Class prep] Facility and equipment setup, venue arrangement	Oversee preparation (arrange equipment and venue)	X (none)
20 mins	[OT] Volunteer training and briefing (key activity guidelines, student profile check)	Conduct volunteer training (deliver key instructions)	Check matched student profile (review previous activity logs and roles)
5 mins	1. Greetings and warm-up (student arrival)	Program preparation and safety check	Student escort and condition check
10 mins	2. Tempo training and static stretching	Tempo lead, stretching demonstration and guidance	Movement assistance and stretching aid
10 mins	3. Warm-up exercise with handball	Conduct warm-up, encourage participation	2:1 Student-volunteer matching, individualized guidance and support
15 mins	4. Main activity 1 (handball basic skills station)	Station supervision and individualized field feedback	2:1 Student-volunteer matching, individualized guidance and support
15 mins	5. Main activity 2 (handball basic skills team game)	Team formation, game rule explanation and management	2:1 Student-volunteer matching, individualized guidance and support
5 mins	6. Cool-down	Cool-down demonstration and guidance	Cool-down assistance
5 mins	7. Activity feedback (For Students)	Praise and encourage students	Encourage students and wrap-up activity
5 mins	8. Greetings and session end (student departure)	Final greetings and safe dismissal	Escort to classroom
30 mins	[Wrap-up] volunteer training and feedback (activity log writing, activity feedback)	Provide additional training (feedback on student specifics)	Write matched student activity log (record specifics and listen to feedback)

### 2.4 Data analysis

All collected data were analyzed following the six-phase procedures of inductive thematic analysis proposed by Braun and Clarke (2006). In Phase 1 (Familiarization), all interview transcripts and field notes were read repeatedly to gain an overall sense of the data. In Phase 2 (Initial Coding), meaningful text segments were extracted and labeled with descriptive codes (e.g., “volunteer initial fear,” “student skill improvement,” “teacher rediscovery of potential”). In Phase 3 (Theme Development), codes sharing conceptual relevance were grouped into candidate sub-themes. In Phase 4 (Reviewing Themes), sub-themes were reviewed against the full dataset to confirm accurate representation. In Phase 5 (Defining Themes), three core themes were finalized with explicit reference to the research questions. In Phase 6 (Writing Up), the analytical narrative was composed. The collected textual data were organized manually using Microsoft Excel 2021 (Microsoft Corporation, Redmond, WA, USA); the spreadsheet served as an organizational tool for sorting and labeling meaning units, not as qualitative analysis software. This process resulted in 39 meaning units, 8 sub-themes, and 3 core themes (Table 4). After the themes were derived, a peer review was performed by two colleagues specializing in adapted physical education (APE) who have conducted qualitative research in the field (one faculty member and one doctoral researcher), alongside an iterative process of reading and refining the entire dataset for further precision.

**TABLE 4:** Summary of inductive thematic analysis results.

Core theme	Sub-theme	Meaning units
Cultivating the ground	Finding seeds in barren land: planning process 1	Identifying blind spots; utilizing corporate sports assets; identifying social needs; moving beyond simple volunteering; designing a strategic CSR model
	Removing stones and leveling the soil: planning process 2	Trust in professional instructors; establishing a cooperation system; preventing safety accidents; qualitative improvement of program standards
	Fertilizing and nurturing: execution process	Institutional mechanisms; importance of pre-education; repetition and continuity
Blooming the flowers	Sprouting the stems of growth: students with developmental disabilities	Rules and routines; interaction; communication skills and sociality; success experience; physical self-efficacy; self-esteem
	Seeing the flowers in full bloom: special education teachers	Student potential; how to use teaching tools; pedagogical inspiration; rediscovery; resolving labor shortage; satisfying the need for dynamic physical education
Harvesting the fruit	Fruit of coexistence with the shell peeled: laying the foundation for successful inclusive physical education	Resolving ambiguity and fear; achieving common goals; sense of immersion; importance of accumulated experience
	Ripening fruit of reflection: healing and reflection	Understanding; sense of pride; feeling rewarded; reflection on communication style; self-esteem; vitality
	Abundant harvest gathered together: team building and organizational pride	Sense of belonging; company loyalty; pride; sense of bonding; consensus

### 2.5 Ensuring research trustworthiness

To ensure the trustworthiness of this qualitative study, several measures were taken based on the criteria proposed by Lincoln and Guba (1985). First, to enhance credibility, triangulation was employed by utilizing diverse data sources, including participant observation and in-depth interviews, and a member check was conducted to verify the analysis results with the research participants. Second, to ensure transferability, the research background, participants, procedures, and context were described in thick detail. Third, to maintain dependability and confirmability, continuous consultations were held throughout the study with two peer researchers — a faculty member and a doctoral researcher, both with qualitative research experience in APE— to ensure that the research process and flow remained traceable.

## 3 Results and discussion

This study aimed to explore the planning and execution process of CSR activities based on APA and the experiences of various stakeholders through an action research methodology. By analyzing the collected data using inductive thematic analysis, a total of 39 meaning units, 8 sub-themes, and 3 core themes were finally derived. The themes identified for each research question are presented in Table 4.

### 3.1 Cultivating the ground: strategic planning and cross-sector collaboration

The first research question regarding the planning and execution of social contribution activities is encapsulated in the overarching theme, “Cultivating the Ground.” This signifies the process wherein the corporation departed from conventional CSR practices to pioneer a new content area, adapted physical activity, and cultivated the soil through cooperation and trial and error, much like the labor and sincerity required of a farmer tilling a field.

#### 3.1.1 Finding seeds in barren land: planning process 1

The program planning originated from the corporation’s in-depth investigation. Participants 22 and 23, from the corporate CSR team, mentioned in their interviews that they focused on identifying “blind spots,” which are areas neglected by public interest but in need of social illumination. Furthermore, they sought to actively utilize the corporation’s existing sports asset: its professional handball team.

We started with the idea of exploring areas that others don’t often touch. While systems have improved, we viewed developmental disabilities as a blind spot in social welfare. We believed physical activity could be a great way to intervene in students’ developmental processes before functional stagnation… We also confirmed through our research that special schools wanted physical activity programs but struggled due to a lack of on-site manpower (Participant 22).

We wondered how to best use our resources and thought of our sports team. Since the company has a handball team, we brainstormed how to support the health of students with developmental disabilities. We wanted a proper curriculum, so we reached out to the Department of APE at K University to request a customized and optimized program (Participant 22).

By combining its internal asset (the handball team) with the social demand for physical activity support for students with developmental disabilities, the corporation envisioned a new field-based CSR model for special schools. This represented an attempt to secure CSR authenticity by investing core corporate competencies into solving social problems, moving beyond simple philanthropic donations.

#### 3.1.2 Removing stones and leveling the soil: planning process 2

While the corporation possessed financial and human resources, it faced limitations in managing the specialized field of APA solely with internal capabilities. To compensate, it established a partnership with K University’s Department of APE, recruited local special schools as program sites, and built an operational system in collaboration with a professional non-governmental organization (NGO) specializing in disability welfare and volunteer program coordination, to increase administrative efficiency. Participants cited this expert collaboration system as a key factor in the program’s successful settlement. In particular, special school teachers expressed high trust, noting that the deployment of verified instructors from a higher education institution ensured the quality and safety of the program.

Knowing that instructors specialized in that field would lead the instructions relieved my burden and concerns; I participated with great expectations (Participant 30).

The program system seems very stable. They anticipate concerns from teachers or parents, maintain individual student logs, and conduct volunteer orientations every time (Participant 29).

These accounts from teachers highlighted that deployment of credentialed APA specialists directly addressed the trust deficit that special school staff typically experience when external programs are introduced (Oh, 2008).

Because they specialized in APA, the entire process, from warm-ups and stretching to various drills, felt very systematic. It was impressive how they created activities using the equipment already at our school, allowing the kids to practice and eventually synthesize everything they learned (Participant 28).

This program is very well-planned and structured… From the planning stage, it is a model that generates social impact through systematic collaboration with expert groups, such as the corporate sports team and K University (Participant 22).

Lee (2014) noted that a lack of expertise and dedicated manpower are major internal factors hindering corporate CSR activities. Therefore, collaborating with local organizations that possess the required expertise is an effective strategy for ensuring the quality and stability of CSR models. The handball program in this study achieved systematization and stabilization through three complementary mechanisms: (1) K University’s APA expertise ensured a structured, pedagogically sound curriculum aligned with students’ developmental needs; (2) the NGO provided administrative infrastructure including scheduling, volunteer coordination, and documentation systems; and (3) Company S’s institutional commitment—mandatory volunteer hours and a dedicated volunteer portal—ensured continuity of human resources across cycles. Together, these mechanisms created the conditions for stable and replicable program delivery (Lee, 2014).

#### 3.1.3 Fertilizing and nurturing: execution process

During the execution phase, the program’s sustainability was anchored in the corporation’s volunteer culture and institutional frameworks. Mandatory annual volunteer hours and designating specific programs as “essential” served as a “priming water” to encourage initial participation. However, this external motivation transformed into voluntary engagement through on-site experience. The process revealed that understanding the value of one’s participation and receiving continuous motivation were highly effective.

We have a dedicated volunteer website. The system shows how many hours I’ve volunteered this year and calculates the social value I’ve created in monetary terms. There are about ten programs, and the developmental disability theme is a “mandatory volunteer” activity requiring participation at least twice a year. Since these involve scheduled face-to-face interactions, they are managed as essential programs (Participant 22).

As you said, while I only come once or twice, this time could be incredibly precious for this student… I felt a great sense of responsibility; if I treated this as just a mandatory task, I would be taking away that student’s opportunity (Participant 3).

I believe the competency of the program instructor has the greatest influence on volunteer re-participation. As a manager, my biggest concern is that volunteers feel they’ve wasted their time. Even if it’s a simple volunteer task, if they feel they weren’t helpful after taking time off work, they don’t apply again. I’m grateful to the instructor for constantly reminding us why we do this and how precious this time is for the children. I think that kind of training is crucial and very helpful (Participant 23).

Providing corporate members with a sufficient understanding of and motivation for CSR activities is vital for shifting participation from mandatory to voluntary and learning-oriented, serving as an essential foundation for the successful settlement of CSR programs (Hyun and Kim, 2010). In this study, the role of professional APA instructors, who conducted pre-volunteer training and managed the sessions, was instrumental in building this foundation.

### 3.2 Blooming the flowers: psychological growth and educational impact for students and teachers

The second research question, regarding the significance in the special school field, resulted in two themes: “Sprouting the Stems of Growth” from the student perspective and “Seeing the Flowers in Full Bloom” from the teacher perspective.

#### 3.2.1 Sprouting the stems of growth: developmental changes in students as observed by teachers and volunteers

This sub-theme focuses on the developmental changes experienced by students with developmental disabilities as observed through teacher reports and field journal entries. It is important to note that, given the communicative characteristics of the student participants, the student perspective is necessarily represented through teacher observation rather than direct student voice. All participant quotations in this section are therefore drawn from special education teachers (Participants 26, 28, 29) and corporate volunteers (Participant 10) who directly observed student behavior across program sessions, rather than from the students themselves. This methodological constraint is consistent with established practice in research involving participants with developmental disabilities.

For students with developmental disabilities, participating in weekly physical activities with new corporate volunteers was a significant challenge (Shields and Synnot, 2016). However, “sports” served as an active medium, helping students accept unfamiliar stimuli as joy and naturally learn how to communicate with the world (Sherrill, 2004). In particular, handball, as a team sport, functioned as highly effective content because it naturally involved diverse interactions within every unit of activity. Throughout the handball program, various educational effects emerged as students’ experiences accumulated in multiple ways.

Children are currently in a period of active hormonal changes and rapid development. At this time, they need to experience various ways to release energy or stress and feel the sensation after sweating through physical activity. Also, our kids usually only meet the same people: family, teachers, or activity assistants. While they might not always meet people as kind as the volunteers, I think experiencing and filling gaps in their relationships through new people is significant. There is much more value here than simply improving handball skills (Participant 26).

Participant 26’s observation captures the relational dimension of APA participation: beyond physical skill development, structured sporting interactions provided students with rare opportunities to engage with unfamiliar adults in a positive, goal-directed context (Allport, 1954).

It can be seen as a time for children to build emotional bonds with people they are meeting for the first time. I believe this process serves as a catalyst to gradually reduce the distance for students who are highly wary of outsiders. Moreover, as it involves physical contact and eye contact to exchange the ball, those elements are naturally embedded in the program. Beyond just play, exercise, or learning new movements, it is an excellent program that involves experiencing the process of building rapport with new people… While I cannot perfectly represent the children’s feelings, seeing their expressions when they meet the volunteers makes me think they might have felt, “I can feel good and happy even when meeting new people, not just those I see every day.” More value could be placed on that aspect of this program (Participant 28).

At first, I was very worried about Student C, but lately, C joined the warm-up exercises (laughs). Seeing C try this and that as the experience accumulated was impressive. Because other teachers reacted with “Wow, C, that’s amazing,” the child seems to be challenging the activities more. The mother mentioned C has low self-esteem, so trying new things might have been difficult, but with the instructors’ cheers and encouragement, I felt C was moving forward step by step while adapting to the program (Participant 29).

As program sessions accumulated, students showed improvements in handball techniques and independent task-solving. These developmental milestones can be interpreted through Bandura’s (1977) four sources of self-efficacy. Most prominently, enactive mastery experience—the most potent source—was evident in students’ progressive skill acquisition (e.g., Participant 10’s observation of marked improvement in throwing form and concentration across sessions). Verbal persuasion, the third source, was actively provided through instructors’ and teachers’ encouragement: Participant 29 noted that the student “seems to be challenging the activities more” in direct response to praise. Additionally, vicarious experience was available as students observed peers successfully engaging in activities. These converging efficacy sources facilitated generalized behavioral changes—such as independent self-preparation for sessions (Participant 28)—reflecting broader social adaptation consistent with the aims of social inclusion (Sherrill, 2004; Shields and Synnot, 2016).

The improvement I felt between the first and second time I saw Student D was truly surprising and encouraging. Honestly, at first, I wondered if the kids could learn this. I thought perhaps the significance lay simply in participating in physical activity. However, by the second meeting, their skills had improved so much. It was amazing to realize that learning was actually happening. The throwing motion itself had improved significantly, and concentration on the activity was much better than before. Since students would have practiced those activities during the intervening weeks, I believe the continuous repetition led to this improvement (Participant 10).

Is it called a Uni-bar? We practiced jumping forward when it was placed horizontally and sideways when placed vertically. But later, I saw the kids doing it on their own during other times. They learn various body movements from the instructor here and show them elsewhere. They gradually learn to move according to instructions, such as jumping sideways, running fast, or walking slowly, and they memorize the stretching motions. At first, they just mimicked the movements, but now they perform them perfectly. I think the kids learned a lot in that regard (Participant 28).

I am really grateful to the students because when I tell them the schedule in the morning, “Hey guys, today is handball day,” they remember it. After other classes, they take out their clothes in advance and change on their own. Honestly, it’s not easy for our kids to do everything themselves, but because they have to go to handball class, they change clothes, store them, and change back afterward. In that sense, it was helpful for daily living skills training. They understood and acquired new rules without refusing them (Participant 28).

This account illustrates not only the transfer of learned movements to other contexts—a hallmark of skill generalization—but also the internalization of structured routines. From a self-efficacy perspective (Bandura, 1977), the student’s ability to reproduce movements independently and follow new rules signals the consolidation of mastery experience into stable behavioral self-regulation.

#### 3.2.2 Seeing the flowers in full bloom: special education teachers

The difficulties special education teachers face in approaching PE classes are among the primary issues repeatedly raised in special school settings, with a lack of manpower, space constraints, and a lack of specialized expertise in PE often cited as the main causes (Goo and Lee, 2016; Oh, 2008; Yoon and Park, 2022). For teachers in these situations, this program served as a catalyst for expanding their educational horizons “broader and further” beyond physical limitations.

The biggest strength of this program is the 1:1 or 2:1 matching between volunteers and students. In a typical school setting, one teacher is forced to handle multiple students, even though each individual student needs help. Even with a high-performing student, if I focus on one, the others just stand around blankly even if I give them other tasks. I always felt bad about that, but the handball class filled that gap. I participated last year too, but there were so many moments where I thought, “Wow, our student can do this?” Because of this intensive 1:1 interaction, I honestly think they showed much higher achievement compared to the level I expected as a teacher (Participant 29).

By observing students engage actively in the program through interactions with volunteers every week, teachers reported experiences of rediscovering students’ potential and expanding their understanding of them. Furthermore, they mentioned gaining hints for effective APA approaches by watching the professional APA instructor organize activities using various equipment already stored at the school.

Oh, Student E can do this too? I learned that if we do it this way, these things are possible and that E likes these activities. There were moments like that where I learned more about E’s abilities. And I had no idea Student F liked and handled the ball so well. Watching them, I thought about other things we could try if that was possible. Student G also had better physical abilities than I thought. I didn’t think G would participate well because of sensory sensitivity, but G did great. It really was a rediscovery of the students (Participant 26).

Even though I graduated from the special education department, it’s really difficult to cover all subjects sufficiently… Space is also an issue; the gym is always fully booked, and liability concerns make outdoor options difficult… I eventually give up on PE. But this time, the children were exposed to handball… and I saw how they set up and use the equipment, thinking that since this is all our school’s equipment anyway, I should try using it like that. It was so interesting that I even took pictures to try it myself next time (Participant 26).

Teachers expressed high satisfaction in being able to provide dynamic PE—active, movement-intensive instructional approaches involving varied and progressively challenging tasks, associated with improved motor development and social engagement in students with disabilities (Block, 2016)—classes that were previously difficult to access due to manpower shortages and space constraints (Oh, 2008). They also identified this as a fundamental starting point where various changes could occur. This case suggests that the provision of corporate human resources and the deployment of professional APA instructors can serve as a practical alternative to fill chronic deficiencies in the public education sector and increase the accessibility and effectiveness of physical activities.

### 3.3 Harvesting the fruit: psychosocial benefits and organizational value for corporate volunteers

The third research question, concerning the significance of APA-based social contribution activities for corporate members, is summarized as “The Value of Being Together.” This encompasses a two-way value creation process that goes beyond simple philanthropic volunteering; through these activities, corporate members personally experience diverse values and develop a sense of organizational pride.

#### 3.3.1 Fruit of coexistence with the shell peeled: laying the foundation for successful inclusive physical education

Research participants noted the unique power of “sports” as a content medium compared to other volunteer activities they had experienced (Sherrill, 2004). Even when students faced challenges in verbal communication, the process of running, sweating, and striving together toward a common goal naturally facilitated various forms of interaction (Allport, 1954). As rapport was built, psychological barriers were rapidly dismantled. These experiences naturally fostered a virtuous cycle, leading to a deeper understanding of disabilities and a motivation for continued participation.

The testimonies from volunteers demonstrate that the APA-based CSR program not only possesses expert leadership and an effective support system but also positively influences the formation of perceptions among non-disabled individuals. This is evaluated as a significant empirical case that has successfully established the essential foundation for moving toward the “ideal unified sports” model proposed by Park and Kim (2011).

Because it involves physical activity together, it feels easier to approach. You get close quickly when you exercise together, right? I think that’s why these students opened up their hearts much faster. I was surprised that I was able to communicate with that student after just three visits. Honestly, I wondered if I could ever have a conversation with him even if we spent a year together… but running in the same direction with a single goal while sweating together is truly powerful. Dribbling the ball and shooting toward the goal together are the kinds of activities that are incredibly helpful in that regard (Participant 8).

This account directly reflects Allport’s (1954) condition of cooperative interdependence: the shared physical goal created a common experiential space that rapidly dismantled initial psychological distance, consistent with Pettigrew and Tropp’s (2006) finding that cooperative, goal-directed contact is especially effective at reducing prejudice.

I used to go to volunteer programs for children with developmental disabilities periodically, mostly just walking in the park. But this program definitely felt more like “education.” I don’t mean that other things are meaningless, but in this program, I could see that these students have a learning process and can improve. It was fascinating to feel that they, too, have a desire for self-improvement. Since that’s a fundamental human motivation, right? It was amazing to see that in them. Also, here, we talk a lot while passing or shooting. In the previous walking activities, they had more independent tendencies, so my words and their reactions were limited, but here, there was much more interaction. I personally like sports where you can play with someone else, and I believe a bond is formed through such activities. Even though communication is difficult, seeing that kind of bond form in such a short time was very meaningful (Participant 10).

Participant 10’s comparison with passive walking activities underscores the unique value of APA as a volunteer medium: unlike non-interactive forms of volunteering, structured sport demanded mutual engagement and sustained interaction, generating precisely the kind of interpersonal familiarity and cooperative experience that facilitates genuine intergroup attitude change (Pettigrew and Tropp, 2006).

I realized I had been living with so many walls… if communication doesn’t work well, we can just communicate in whatever way works. After experiencing this, I felt it was a quite good experience and thought that I could definitely come back next time (Participant 3).

Participant 3’s reflection illustrates the psychological barrier reduction that Allport (1954) identified as a central outcome of successful intergroup contact. The willingness to return for future participation further suggests that the contact experience generated positive intergroup attitudes that extended beyond the immediate session—a pattern consistent with Pettigrew and Tropp’s (2006) finding that accumulated contact experiences produce increasingly durable reductions in prejudice. Taken together, the accounts of Participants 8, 10, and 3 confirm that the APA-based program created the conditions necessary for meaningful intergroup contact, functioning as an empirical instance of Allport’s (1954) contact hypothesis in action.

#### 3.3.2 Ripening fruit of reflection: healing and reflection

Most participants expressed that while they held a vague sense of fear prior to the activity, their participation served as an opportunity to expand their understanding of disabilities (Pettigrew and Tropp, 2006). This awareness became more pronounced as experiences accumulated through repeated participation. Furthermore, volunteers who participated in the program two or more times experienced various positive emotions, defining their volunteer time as a “time for healing and reflection.”

At first, this student kept talking to himself, and I didn’t understand what he was saying… But after meeting him two or three times, I finally began to understand… Once I realized that, I decided to communicate in his way, and he loved it. That’s when it hit me: “I shouldn’t communicate only in my way; I need to talk with him in his way…” There is a sense of pride and fulfillment that comes from knowing I was helpful and did something good (Participant 8).

I think many people feel similarly. Humans naturally feel happiness in the process of helping others and making them happy, don’t they? Doing a volunteer activity like this program, which feels like it provides substantive help to the children, remains a source of pride. It’s also meaningful to be able to think more deeply about children with developmental disabilities… I don’t think I’m the only one who feels this way (Participant 9).

These accounts illustrate that repeated participation in the APA-based CSR program generated affective outcomes extending beyond task completion — including a deepened understanding of disability, a sense of personal fulfillment, and motivation for continued engagement. This is consistent with Pettigrew and Tropp’s (2006) finding that accumulated intergroup contact produces increasingly positive attitudes over time, and with Hyun and Kim’s (2010) finding that meaningful CSR participation fosters a sense of contribution and satisfaction among volunteers.

#### 3.3.3 Abundant harvest gathered together: team building and organizational pride

In modern society, corporations have taken on the characteristics of power entities that exert significant political and social influence, in addition to being economic actors (Carroll and Shabana, 2010). Consequently, the concept of “Corporate Citizenship” has become established, defining corporations as members of society and assigning them corresponding rights and duties (Matten and Crane, 2005). In this study, we observed volunteers progressing toward becoming healthy corporate citizens who exercise positive influence across society through the CSR program.

Externally, when it becomes known that our company isn’t just making money but is solving social problems in unseen places and creating various values, I believe an intangible energy, like pride among members that they work for such a company and directly contributed to that process, is generated. I think this will manifest as a positive effect internally as well (Participant 22).

As a large corporation, if we earn money through society, I believe it’s only natural to give back to marginalized areas… Companies go through booms and busts, but even in difficult situations, I feel even more pride that we continue these activities… That kind of corporate image is one of the reasons I want to stay with this company (Participant 8).

Both accounts reflect the internalization of corporate citizenship values (Matten and Crane, 2005): organizational pride is not passively received but actively constructed through the experience of witnessing one’s company fulfill its social responsibilities in practice. Participants also experienced a “team-building” effect, gaining a more human understanding of their colleagues by volunteering together and strengthening bonds among corporate members (Tosti and Stotz, 2001). They expressed significant pride in the corporation’s commitment to sincere social contribution (Kim et al., 2018).

I believe a bond is formed by sharing the same experience… Through this activity, we share a similar experience and talk about how we felt… Seeing those sides allowed me to discover hidden strengths in them. Back at the office, I think differently now… It’s socially helpful and has promotional effects, so there’s no reason for the company not to do it (Participant 10).

Our organization has 70 people with various ages and ranks… Since everyone does their own work, even a team of about 10 people doesn’t have many topics to talk about together. But by participating like this… we naturally talk about the same subject… You can see people’s personalities by how they approach the activity. I saw new sides of friends in the same organization whom I rarely had the chance to talk to. Understanding those new sides to them was also very good (Participant 8).

A method where members actively participate in solving social problems and co-creating value, rather than a top-down approach led unilaterally by the corporation, has a powerful motivational effect (Porter and Kramer, 2011). This can satisfy the needs of members by providing values such as job satisfaction, organizational commitment, and increased company loyalty (Kim et al., 2018). In this regard, the program conducted in this study is evaluated as an effective model that embodies the core values pursued by successful CSR models.

### 3.4 Comprehensive discussion: a virtuous cycle model of APA-based CSR

Within the specific context examined, the findings suggest that corporate CSR activities based on APA show potential as a multi-directional value-creation approach (Porter and Kramer, 2011) and provide an empirical instance of Allport’s (1954) Intergroup Contact Theory in action. Notably, the program fulfilled three of Allport’s (1954) four optimal conditions for prejudice reduction: (1) common goals—volunteers and students worked cooperatively toward shared handball objectives; (2) intergroup cooperation—the 2:1 volunteer-to-student matching structure required sustained collaborative interaction; and (3) institutional support—Company S’s mandatory volunteer framework and K University’s professional oversight provided authoritative sanction for the contact situation (Pettigrew and Tropp, 2006). The condition of equal status was partially met; while volunteers and students occupied different social roles, the physical activity context created a shared experiential space that mitigated hierarchical distance, consistent with Pettigrew and Tropp’s (2006) finding that Allport’s (1954) conditions are facilitating rather than strictly necessary.

Based on the results of this action research on APA-based corporate social contribution activities, the comprehensive discussion can be summarized as follows:

First, the excellence of APA as a content medium: Physical activity functioned as a universal language that transcends linguistic barriers. The physical contact and cooperation between students with disabilities and corporate volunteers triggered by sports effectively shortened the time needed to build rapport, eliciting an emotional connection that is difficult to experience in other types of volunteer work (Sherrill, 2004; Allport, 1954).

Second, the importance of a collaborative structure backed by expertise: The quality of the program was elevated by addressing field demands, which are difficult to resolve through corporate capital and will alone, through collaboration with expert groups (Lee, 2014). This provided volunteers with a sense of efficacy, knowing they were offering substantive support as participants in a professional program, and gave teachers confidence in the safety and systematic nature of the classes (Oh, 2008).

Third, organizational performance within the corporation: Moving beyond simple philanthropic activities, this initiative created internal branding effects by providing members with opportunities for healing and reflection while inspiring mutual understanding among colleagues and pride in the organization (Tosti and Stotz, 2001). This suggests that CSR applying appropriate content can be interpreted as an effective investment in intangible values that goes beyond social contribution through simple expenditure in terms of sustainable corporate management.

## 4 Conclusion and suggestions

This study explored the planning, execution, and psychological impacts of an APA-based CSR program through an action research approach. Moving beyond traditional philanthropic models, this cross-sector collaborative initiative demonstrated how combining corporate resources with APA expertise can contribute to building a more inclusive educational environment within the specific contexts studied.

The findings confirm that the program functioned as a powerful catalyst for multidimensional psychological and developmental growth among all stakeholders. These findings are broadly consistent with prior research demonstrating the psychosocial benefits of APA participation for individuals with disabilities (Sherrill, 2004; Shields and Synnot, 2016) and with CSR literature showing that employee volunteering enhances organizational commitment and pride (Kim et al., 2018; Tosti and Stotz, 2001). However, this study extends these bodies of work in two important respects. First, unlike prior APA studies that evaluate outcomes primarily from a physical development standpoint, this study foregrounds the relational and psychological dimensions, linking them explicitly to Bandura’s (1977) self-efficacy framework. Second, unlike most CSR-volunteering studies that focus on corporate-side outcomes, this study provides a simultaneous multi-stakeholder account—offering a more comprehensive understanding of shared value creation consistent with Porter and Kramer’s (2011) CSV model. For students with developmental disabilities, structured physical activities served as an effective medium to enhance physical self-efficacy and facilitate social interaction. For special education teachers, it expanded their educational horizons by providing practical experiences with dynamic APA methodologies. Most notably, for corporate volunteers, the process of sweating and cooperating together naturally facilitated intergroup contact, which rapidly dismantled psychological barriers, reduced ableism, and ultimately fostered a strong sense of organizational pride and corporate citizenship.

Several limitations of this study warrant explicit acknowledgment. First, the researcher’s dual role as program instructor and primary analyst introduces a risk of confirmation bias. While constant reflexivity was employed throughout (Finlay, 2002), the resulting analysis may disproportionately reflect positive outcomes. Certain ambivalent experiences were observed in the field—some volunteers reported initial discomfort that was difficult to articulate, and a small number of students exhibited resistance during early sessions—yet these did not develop into major analytic themes due to their limited frequency in the data. Future studies should deliberately elicit and analyze challenging or contradictory experiences. Second, this study is based on a single organizational case (Company S) conducted in two special schools within one city, which limits transferability. The findings should be understood as exploratory and hypothesis-generating rather than definitive. Third, the absence of direct student voice—due to the communicative characteristics of the participants—limits the completeness of the student perspective, which was largely mediated through teacher observation.

Based on these findings, the following suggestions are made for future research and practice. First, to solidify the psychological mechanisms identified in this qualitative study, future research should conduct mixed-methods or longitudinal quantitative studies that objectively measure changes in variables such as students’ physical self-efficacy, volunteers’ prejudice reduction, and organizational commitment. Second, to ensure the long-term sustainability of such inclusive models, institutional support systems—such as creating manuals for APA-based CSR operations and establishing matching platforms between corporate ESG teams and local special schools—must be developed. Ultimately, it is hoped that this triadic collaboration model will be widely adopted as a standard practice for inclusive education and corporate shared value creation.

## Data Availability

The raw data supporting the conclusions of this article will be made available by the author, without undue reservation.
